# Meniscotibial ligament pockets on MRI are associated with radiographic severity of medial knee osteoarthritis

**DOI:** 10.1038/s41598-026-52184-5

**Published:** 2026-06-02

**Authors:** Hisako Katano, Nobutake Ozeki, Yusuke Nakagawa, Hideyuki Koga, Chiaki Okumura, Noriya Okanouchi, Jun Masumoto, Ichiro Sekiya

**Affiliations:** 1 Center for Stem Cell and Regenerative Medicine, Institute of Science Tokyo, Tokyo, Japan; 2 Department of Joint Surgery and Sports Medicine, Graduate School of Medical and Dental Sciences, Institute of Science Tokyo, Tokyo, Japan; 3 Fujifilm Corporation, Tokyo, Japan; 4 Center for Stem Cell and Regenerative Medicine, Department of Applied Regenerative Medicine, Institute of Science Tokyo, 1-5-45 Yushima, Bunkyo-ku, Tokyo, 113-8510 Japan

**Keywords:** Osteoarthritis, knee, Meniscotibial ligament, Meniscal extrusion, Magnetic resonance imaging, Kellgren–Lawrence classification, Epidemiology, Anatomy, Diseases, Medical research, Rheumatology

## Abstract

Meniscotibial ligament dysfunction has been proposed as a factor contributing to medial meniscus extrusion (MME), a key feature of medial knee OA. This study investigated meniscotibial ligament pockets, defined as fluid-equivalent signals on MRI, as MRI findings that may reflect changes at the meniscotibial ligament insertion, including possible detachment. We examined their prevalence across Kellgren–Lawrence (KL) grades and their anatomical distribution. Participants from the Kanagawa Knee Study without lateral knee OA underwent spoiled gradient–recalled MRI. The “pocket sign,” defined as a fluid-equivalent signal between the medial tibial cortex and the ligament attachment on axial and coronal images, was used as an MRI finding potentially related to meniscotibial ligament detachment. Multivariable logistic regression evaluated associations between pocket presence and clinical factors. Pocket location was classified relative to the medial collateral ligament (MCL). A subset underwent surgical assessment during knee arthroplasty. Pocket prevalence increased with KL grade, from 40% in KL grade 0 to 100% in KL grade 4. Higher KL grade was independently associated with pocket presence (OR 2.56 per grade increase, *p* < 0.001). Age showed a limited and inconsistent association, whereas sex and BMI were not associated. In KL grade 0 knees, pockets were predominantly located anterior to the MCL and were associated with mild MME. Surgical findings supported the MRI results. Meniscotibial ligament pockets are present even in KL grade 0 knees and become more frequent with increasing radiographic severity. Their anterior predominance relative to the MCL suggests a characteristic distribution pattern. The pocket sign may serve as an MRI marker of meniscotibial ligament status.

## Introduction

Knee osteoarthritis (OA) is a highly prevalent degenerative joint disease worldwide, with medial knee OA as the most common structural phenotype^1^. Structural alterations involving the meniscus are central to the pathophysiology of medial OA, as the meniscus plays a key role in load distribution, shock absorption, and joint stability^2^. Disruption of meniscal function is associated with cartilage degeneration and the structural progression of OA.

The meniscotibial ligament, also referred to as the coronary ligament, anchors the peripheral margin of the medial meniscus to the tibial plateau to provide meniscal stability^3,4^. Loss of this anchoring function may permit medial meniscus extrusion (MME), which alters load transmission across the joint and is frequently observed in medial knee OA^5,6^. Although meniscotibial ligament dysfunction has been proposed as a structural factor associated with MME, systematic characterization of the in vivo imaging features that reflect ligament attachment status is lacking for population-based cohorts.

We observed a fluid-equivalent signal between the medial tibial cortex and the presumed attachment site of the meniscotibial ligament on MRI. We termed this finding a “meniscotibial ligament pocket” (pocket sign) and defined it as a focal fluid-equivalent signal that directly contacts the tibial cortex at the ligament–bone interface. More proximal equivocal or non-fluid signal was not included in the pocket region. We did not apply a numeric minimum size threshold. Instead, to avoid overdiagnosis, signals identified on axial images were evaluated on the corresponding coronal MPR sections. When the signal appeared localized on the initial coronal section, adjacent coronal sections were reviewed. Signals were classified as positive only when continuity could be confirmed at the same anatomical location across adjacent sections. To minimize misclassification due to single-section artifacts, the pocket finding was required to be visible at the same anatomical location on at least three consecutive MPR sections. This imaging feature is conceptually analogous to the periodontal “pocket,” a term used to describe a space along a tissue interface on imaging^7^. In the present study, the pocket sign was used as a surrogate imaging marker potentially related to meniscotibial ligament detachment, rather than as direct evidence of structural detachment.

The purposes of this study were to determine the prevalence of meniscotibial ligament pockets in a community-based cohort stratified by Kellgren–Lawrence (KL) grade and to examine the anatomical distribution of these pockets, particularly in KL grade 0 knees without radiographic OA. We also evaluated cross-sectional associations between pocket presence and clinical factors, including age, sex, and body mass index.

## Materials and methods

### Kanagawa Knee Study and subject enrollment

This study was approved by the Institutional Review Board (IRB) of the Institute of Science Tokyo (Approval No. M2018-072) and was conducted in accordance with the relevant guidelines/regulations. Informed consent was obtained from all participants, and the study was performed in accordance with the Declaration of Helsinki. The subjects were 573 participants in the Kanagawa Knee Study from the Kanagawa and Tokyo prefectures. Only the right knee was evaluated for each subject^8^. To remove any treatment influences, subjects with a history of knee OA, lower extremity trauma, previous surgery, rheumatoid arthritis, or consecutive visits to one or more hospitals for more than 3 months were excluded. Knee radiographs and MRI scans were performed between September 1, 2018, and August 30, 2019. For the present analysis, subjects with lateral knee OA were excluded^9^.

### Automatic evaluation of KL grade

Radiographs were taken of the anteroposterior view of the knee with the patient in a standing position with full knee extension. Digital Imaging and Communications in Medicine (DICOM) data were sent to the ImageBiopsy Lab, Inc. (Vienna, Austria), and KL grading was automatically performed using the Knee Osteoarthritis Labeling Assistant (KOALA) software (IBLAB-ZOO 1.13.16 KOALA-CE). The grading was reviewed and checked for errors by trained staff at the ImageBiopsy Lab, Inc.; however, to prevent bias, no manual changes were made^9^.

### Exclusion of lateral OA

The following parameters for the Osteoarthritis Research Society International (OARSI) grade were also automatically evaluated using the KOALA software: joint space narrowing, medial (JSM); joint space narrowing, lateral (JSL); osteophytes, tibia medial (OSTM); osteophytes, tibia lateral (OSTL); osteophytes, femur medial (OSFM); and osteophytes, femur lateral (OSFL). Each parameter was graded as 0 (normal), 1 (mild change), 2 (moderate change), or 3 (severe change), with greater values indicating a more severe form of knee OA^10^. To exclude lateral knee OA, subjects were excluded when the JSL was larger than the JSM and the difference between the OSFL and the OSTL was larger than the difference between the OSFM and the OSTM^9^.

### Magnetic resonance imaging

MRI was performed at 3.0 T (Achieva 3.0TX, Philips, Amsterdam, Netherlands) using a dedicated 16-channel transmit/receive knee coil (dStream T/R Knee 16ch coil, Philips). We acquired three-dimensional (3D) sagittal fat-suppressed spoiled gradient–recalled (SPGR) volume sequences in the steady state as well as proton density-weighted (PDW) sequences (Table 1). Coronal and axial images were evaluated as multiplanar reformats derived from the same 3D SPGR volume. The rest time before the MRI scan was approximately 20 min^8^.

**Table 1:** Imaging parameters for MRI sequences.

	SPGR
Repetition time (msec)	20
Echo time (msec)	1st: 7 2nd: 13.8
Flip angles (°)	35
Echo train length	(−)
Acquisition voxel size (mm)	0.6 × 0.6 × 0.6
Reconstruction matrix size (mm)	0.3 × 0.3 × 0.3
No. of slices	320
Slice thickness (mm)	0.3
Slice gap (mm)	0
Field of view (mm × mm)	150 × 150
WFS/BW (pix/Hz)	2.002/217.0
Number of excitations	1
Total examination time	7 min 30 sec

SPGR, fat-suppressed spoiled gradient echo; WFS/BW, actual waterfat shift/bandwidth; MRI, magnetic resonance imaging.

### Detecting meniscotibial ligament detachment–related MRI findings (pocket sign)

**Fig. 1:**
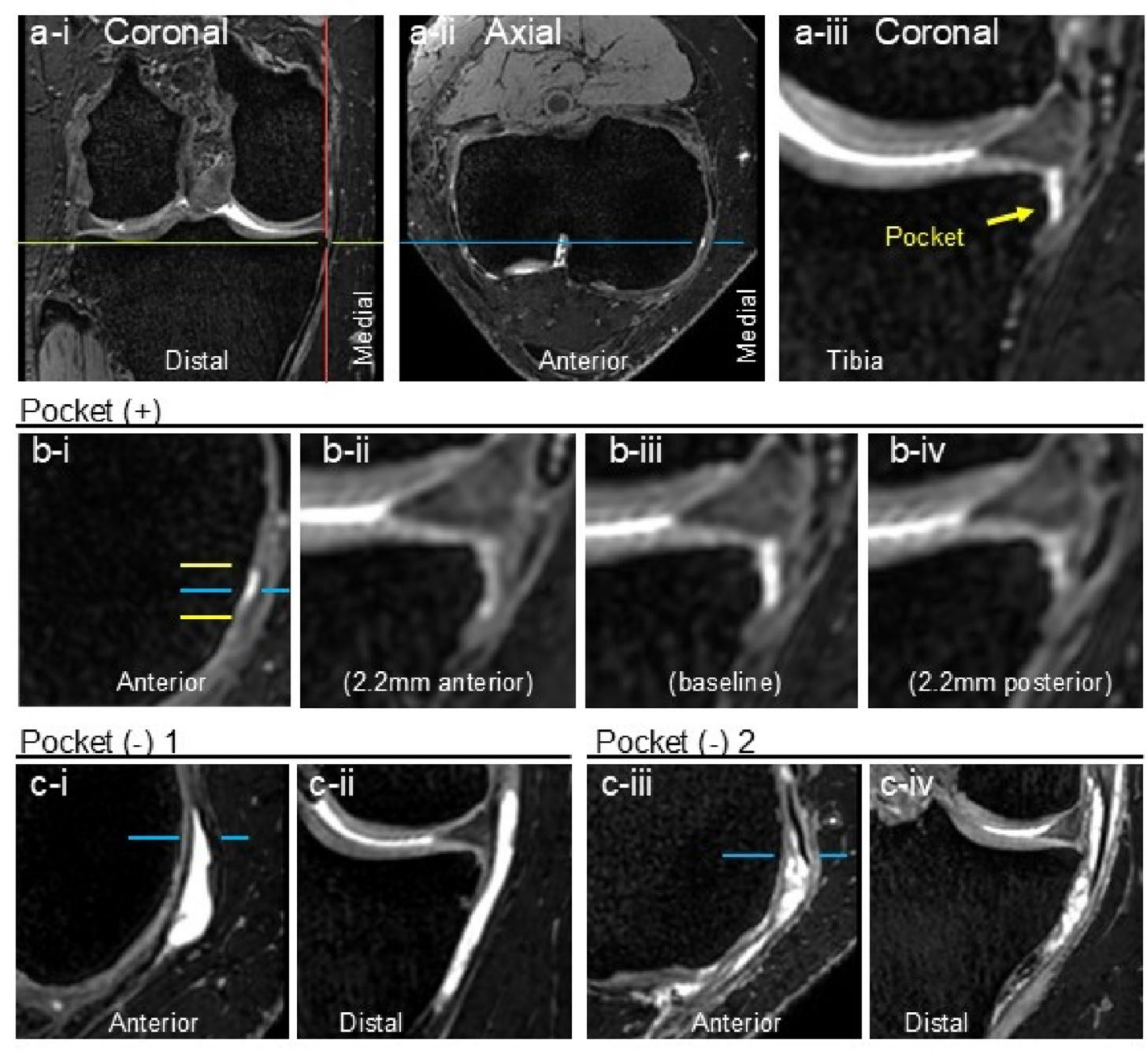
MRI-based method for detecting the pocket sign (a detachment-related finding of the meniscotibial ligament). The pocket sign was defined as a definite fluid-equivalent signal directly contacting the medial tibial cortex at the meniscotibial ligament–bone interface. More proximal equivocal or non-fluid signal was excluded. (**a**) Localization of the evaluation plane. (i) Coronal image at the level of maximal medial tibial plateau width with vertical and horizontal reference lines. (ii) Corresponding axial image used to assess a fluid-equivalent signal around the medial tibial plateau. (iii) Coronal image through the defined level confirming a focal fluid signal at the meniscotibial ligament–tibial interface. (**b**) Representative pocket-positive case. (i) Axial image showing the pocket center (blue line) and corresponding anterior and posterior reference planes (yellow lines). (ii–iv) Coronal images at the pocket center and at the 2.2 mm anterior and posterior levels, demonstrating continuity of the focal fluid signal across adjacent slices. (**c**) Representative pocket-negative cases. (i–ii) Pocket-negative case 1 showing a fluid signal that appears to contact the tibial cortex but is continuous with a fluid collection adjacent to the medial meniscus, consistent with an MCL bursa. (iii–iv) Pocket-negative case 2 illustrating an apparently focal fluid signal at the tibial cortex that, upon evaluation at the same level, is associated with another fluid signal continuous with the medial meniscus and therefore does not meet criteria for pocket positivity.

The pocket sign was evaluated using spoiled gradient–recalled (SPGR) MRI in both axial and coronal planes (Fig. 1). Axial and coronal images were assessed as multiplanar reconstructions (MPR) derived from the same 3D SPGR volume. In these reformats, one-slice increment corresponded to the in-plane pixel spacing (0.29 mm), allowing confirmation of signal continuity across adjacent slices. The pocket sign was defined as a definite fluid-equivalent signal directly contacting the medial tibial cortex at the meniscotibial ligament–bone interface. More proximal equivocal or non-fluid signal was excluded. The pocket region was conservatively limited to definite fluid-equivalent signal directly contacting the medial tibial cortex. Punctate signals were classified as positive only when continuity was confirmed on adjacent slices. No numeric minimum size threshold was applied. Rather than attempting definitive tissue characterization on MRI, structures were included only when they satisfied this signal-based definition.

First, the coronal slice showing the maximal medial tibial plateau width was selected. At this level, the most medial bony contour of the tibial plateau was identified as the reference point. If an osteophyte was present, a vertical reference line was drawn at the most medially projecting distal cortical edge of the osteophyte rather than at the original tibial plateau margin. The distal end of the articular cartilage was also used as an anatomical landmark. The corresponding axial image was then examined for a fluid-equivalent high signal around the medial aspect of the tibia. When this type of signal was detected, the corresponding coronal plane was selected for targeted assessment.

A pocket was defined as the presence of a fluid-like high signal intensity directly contacting the medial tibial cortex at the presumed attachment site of the meniscotibial ligament, but without an intervening low-signal structure between the cortical surface and adjacent fibrous tissue. In knees with osteophytes, direct contact was assessed relative to the cortical surface of the osteophyte. The signal intensity was required to be comparable to that of the joint fluid. Droplet-shaped, linear/band-like, or beaded configurations were considered positive if they were clearly distinguishable from surrounding structures.

To minimize misclassification due to single-section artifacts, the pocket finding was required to be visible at the same anatomical location on at least three consecutive MPR sections. Knees were classified as pocket-negative knees if they had fluid collections consistent with the MCL bursa (typically located between the superficial and deep MCL layers) or dependent joint fluid without clear interposition between the cortical surface (including the osteophyte cortex when present) and ligament attachment.

Knees fulfilling all inclusion criteria and none of the exclusion criteria were classified as pocket-positive knees.

### Reliability assessment

A subset of 50 knees, reflecting the KL grade distribution of the full cohort, was selected for reliability analysis. The MRI scans were independently evaluated by two readers who were blinded to all clinical and radiographic information. For intra-observer reliability, one reader performed a repeated evaluation under blinded conditions. Inter-observer reliability was assessed based on the independent evaluations of the two readers. The observed proportion of agreement and expected agreement were calculated, and Cohen’s kappa coefficient was derived for both intra- and inter-observer reliability^11^. In cases of disagreement within this subset, the images were independently re-evaluated by the first author, and the final classification was based on this determination. For the full cohort (*n* = 469), pocket presence was evaluated by the first author, who established the final dataset used for statistical analysis. Both readers were experienced in musculoskeletal MRI evaluation.

### Multivariable analysis

A logistic regression model was constructed with pocket presence as the dependent variable. KL grade (per 1-grade increase), age (per 1-year increase), sex (male vs. female), and BMI (per 1 kg/m^2^ increase) were included as independent variables. Odds ratios (ORs) with 95% confidence intervals (CIs) were calculated. A p value < 0.05 was considered statistically significant.

### Localization of the pocket sign

MRI data were analyzed using a three-dimensional (3D) image analysis system volume analyzer (SYNAPSE 3D [Japanese product name: SYNAPSE VINCENT], version 6.8; FUJIFILM Corporation, Tokyo, Japan). Bone and meniscus regions were automatically segmented from proton density-weighted (PDW) images, and cartilage regions were segmented from SPGR images. Based on these segmentations, we generated a three-dimensional (3D) reconstruction of the tibial plateau.

Anatomical landmarks used for localization were automatically identified. These included the centroids of the medial and lateral tibial condyles, which defined the reference axis for angular measurement, and the centroid of the entire tibial plateau, which was used as the origin for angular calculation.

For angular measurement, we defined the angle between a line from the tibial center to the relevant medial collateral ligament (MCL) edge and a line from the tibial center to the corresponding pocket edge. If the pocket edge lay between the anterior and posterior edges of the MCL, the value was expressed as negative.

### Medial meniscal extrusion (MME) width at pocket site

We used 3D reconstructions of the tibial plateau and medial meniscus for analysis. The reconstructed model was rotated around the central axis so that the center of the pocket was aligned with the lateral-most position in the viewing plane. This alignment standardized the orientation across subjects and enabled consistent measurement at the pocket site.

We defined the MME width as the horizontal distance from the medial edge of the tibial plateau to the outer margin of the medial meniscus at the pocket site. This measurement was automatically calculated using a frontal 3D view. The values were recorded in millimeters and subsequently categorized into predefined intervals for analysis.

To minimize the influence of osteophytes on automated 3D measurements, we restricted the analysis to KL grade 0 knees with a pocket sign. Because the automated 3D method cannot reliably distinguish bone from osteophytes, limiting the analysis to KL grade 0 knees (without radiographic osteophytes) reduced potential confounding by osteophyte size. Furthermore, as MME was measured specifically at the predefined pocket site, KL grade 0 knees without an identifiable pocket were not eligible for this pocket site–based analysis.

### Intraoperative observations related to the pocket sign

We conducted an intraoperative investigation during total knee arthroplasty in a patient diagnosed with KL grade 3 medial knee OA. A longitudinal medial incision was made in the medial margin to the patellar tendon to expose the anterior horn of the medial meniscus. Gentle distal–medial traction was applied to the anterior horn, and the meniscotibial ligament was carefully dissected from the anterior direction using an electrosurgical device. During this procedure, we macroscopically identified findings at the meniscotibial ligament insertion on the medial tibial plateau, including possible detachment, prior to the completion of the dissection. These findings may correspond to the pocket sign on MRI. This investigation was performed on six knees, and the pocket sign was observed in all cases, providing supportive but limited intraoperative correlation with the MRI findings.

### Statistical analysis

Statistical analyses were performed using BellCurve for Excel (Social Survey Research Information Co., Ltd., Tokyo, Japan). Multivariable logistic regression analysis was conducted to examine factors associated with pocket presence. The dependent variable was pocket presence (yes/no), and independent variables included KL grade, age, sex, and BMI. Odds ratios (ORs) and 95% confidence intervals (CIs) were calculated. Trend analysis across KL grades was performed using the Cochran–Armitage test for trend. Age-stratified analyses were conducted by categorizing age into decades (30s, 40s, 50s, 60s, and 70s). Chi-square tests were used to compare the distribution of pocket locations among groups. A p-value of < 0.05 was considered statistically significant. Inter- and intra-observer reliability were evaluated using Cohen’s kappa coefficients.

## Results

### Participant flow and demographics

**Fig. 2:**
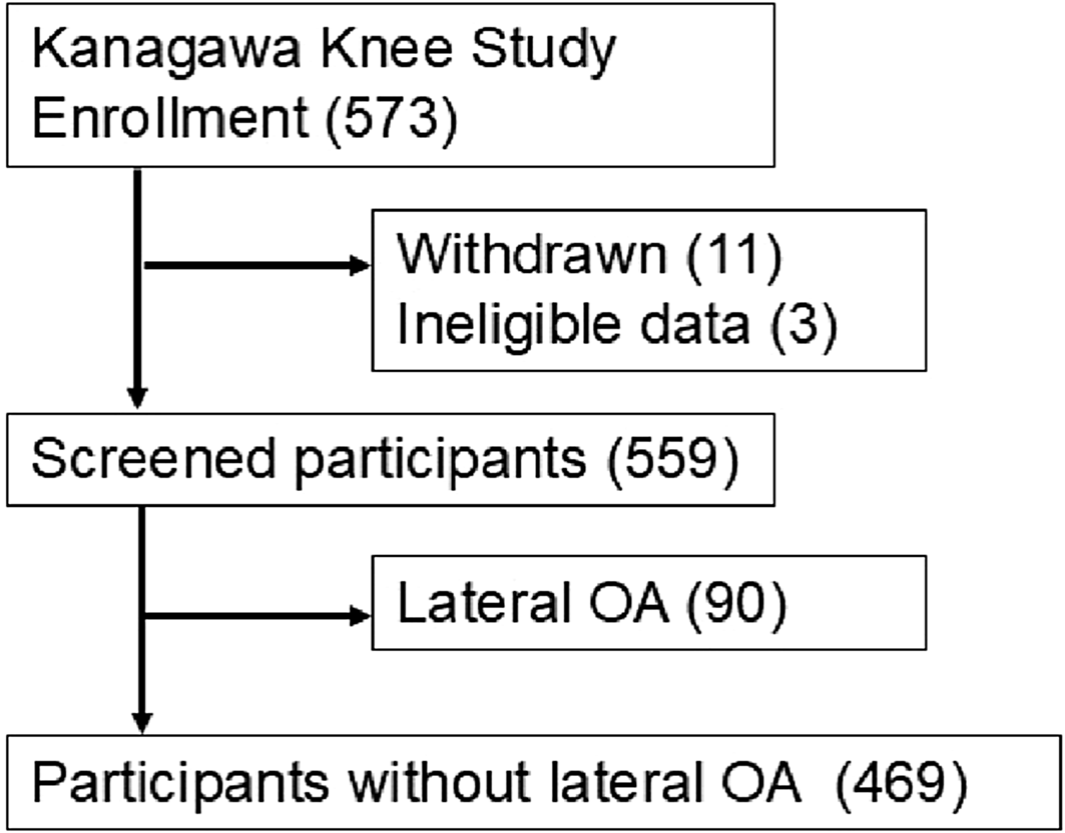
Flowchart for participant selection in the Kanagawa Knee Study. A total of 573 participants were initially enrolled. After excluding 11 individuals who withdrew and 3 with ineligible data, 559 participants were screened. Among them, 90 participants with lateral knee OA were excluded. The remaining 469 participants without lateral knee OA were included in the final analysis.

The Kanagawa Knee Study enrolled 573 participants (Fig. 2). Eleven participants withdrew and 3 had ineligible data, leaving 559 participants who completed screening. Of these, 90 participants with lateral knee OA were excluded. The final study population consisted of 469 participants. Baseline characteristics included a median age of 54 years (range 30–79) and a median BMI of 23 kg/m^2^ (range 16–42) (Table 2). The cohort comprised 241 males (51.4%) and 228 females (48.6%), and most participants were classified as KL grade 0.

**Table 2:** Subject characteristics (*n* = 469).

Characteristic	Value
Age, years	54 (30–79)
BMI, kg/m^2^	23 (16–42)
Sex, n (%)	
Male	241 (51.4)
Female	228 (48.6)
KL grade, n (%)	
KL0	308 (65.7)
KL1	70 (14.9)
KL2	75 (16.0)
KL3	13 (2.8)
KL4	3 (0.6)

Values are presented as median(range) or n (%), as appropriate.

### MRI assessment of meniscotibial ligament detachment–related findings

**Fig. 3:**
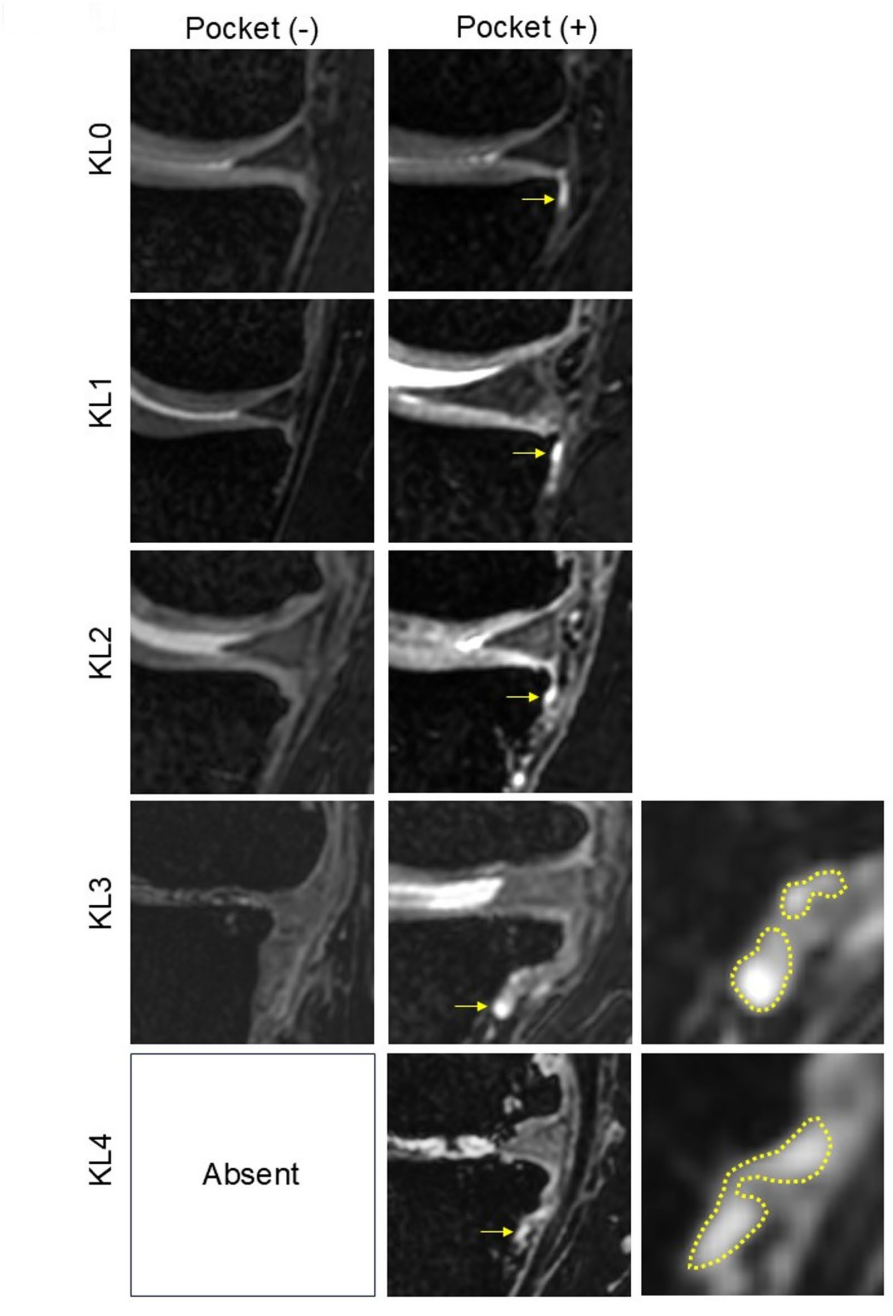
MRI appearance of the pocket sign across KL grades. Coronal SPGR MRI images of the medial compartment show knees without the pocket sign (pocket [−]) and with the pocket sign (pocket [+]) across KL grades. Yellow arrows indicate the characteristic pocket sign, defined as a focal fluid-equivalent signal at the meniscotibial ligament–bone interface. Enlarged images of pocket-positive KL grade 3 and KL grade 4 knees are shown in the rightmost column. In these enlarged images, the region classified as the pocket sign is outlined by a yellow dotted line. The panel labeled “Absent” indicates that no representative pocket-negative knee was available for KL grade 4.

Coronal SPGR MRI images of the medial compartment revealed the presence or absence of the characteristic “pocket” sign (Fig. 3). The pocket sign was observed across all KL grades from KL grade 0 to KL grade 4. In KL grade 0 knees, it appeared as a subtle separation at the meniscotibial junction (yellow arrow). The sign became more pronounced in KL grade 1 through KL grade 4, with a clear separation-like appearance between the ligament attachment and the medial tibial cortex. In contrast, cases without the pocket sign showed preserved apposition of the ligament attachment to the tibial cortex, with no evidence of pocket formation; this was particularly the case in KL grade 0–3 knees. Intra-observer agreement was 88% (44/50 cases), with a Cohen’s κ of 0.74. Inter-observer agreement was 90% (45/50 cases), with a Cohen’s κ of 0.78.

### Multivariable logistic regression analysis for pocket presence

**Fig. 4:**
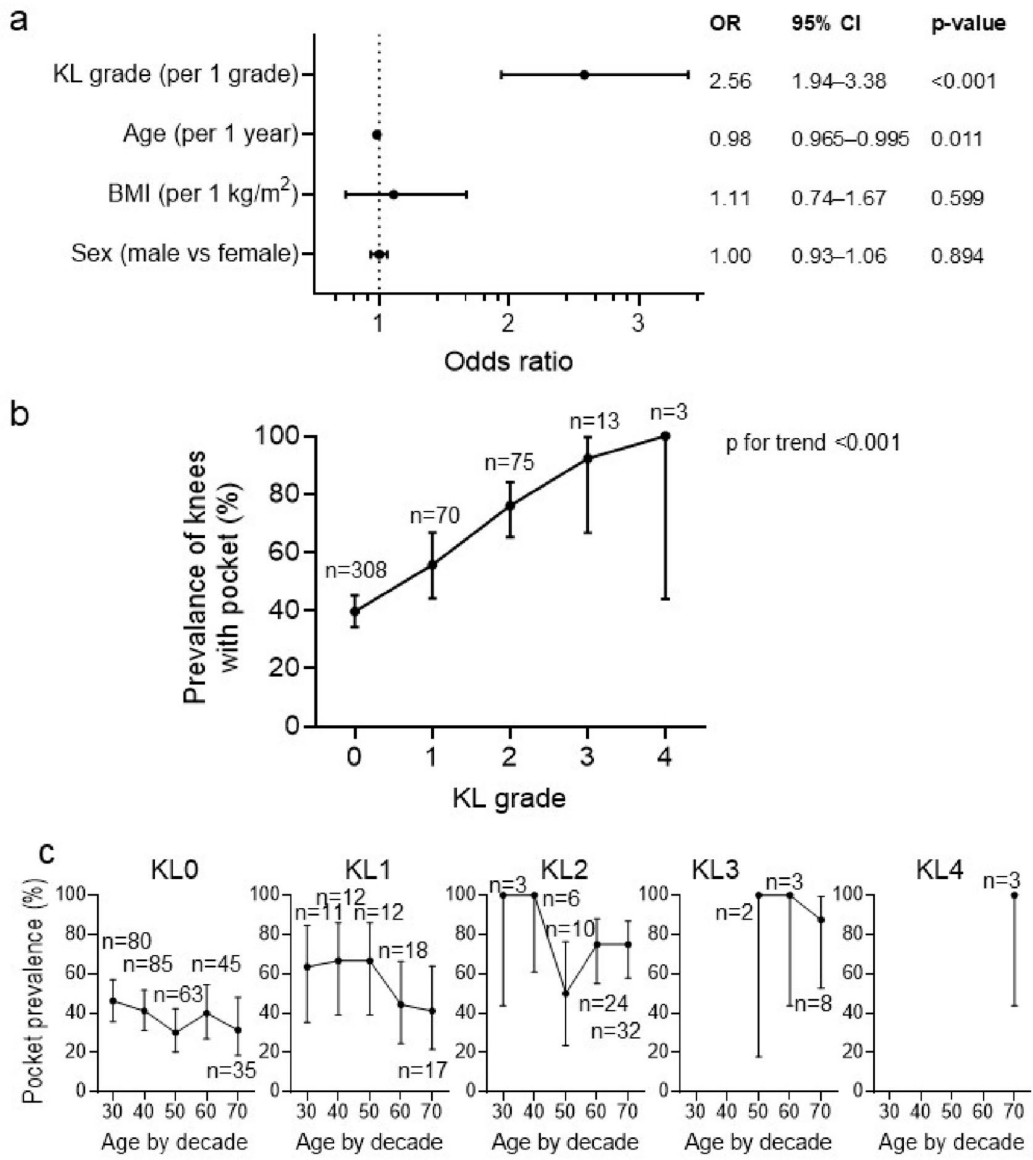
Factors associated with pocket presence. (**a**) Multivariable logistic regression analysis for pocket presence. Odds ratios (points) and 95% confidence intervals (horizontal lines) are shown for KL grade (per 1-grade increase), age (per 1 year), BMI (per 1 kg/m^2^), and sex (male vs. female). The vertical line indicates an odds ratio of 1. (**b**) Prevalence of knees with a pocket sign according to KL grade. Error bars indicate 95% confidence intervals. Sample sizes for each KL grade are shown above the points. A significant trend across KL grades was observed (p for trend < 0.001). (**c**) Prevalence of pocket presence stratified by age in decades within each KL grade. Error bars indicate 95% confidence intervals. The number of knees in each KL group is shown below the plots. No significant association between age and pocket presence was observed within individual KL strata.

A higher KL grade was significantly associated with pocket presence in the multivariable logistic regression model (OR 2.56 per 1-grade increase, 95% CI 1.94–3.38, *p* < 0.001) (Fig. 4). Age was also significantly associated with pocket presence (OR 0.98 per year, 95% CI 0.965–0.995, *p* = 0.011). Sex and BMI were not significantly associated with pocket presence.

### Prevalence of knees with a pocket sign according to KL grade

The prevalence of knees with a pocket sign increased across KL grades. The prevalence ranged from 39% in KL grade 0 to 100% in KL grade 4. A significant trend across KL grades was observed (p for trend < 0.001).

### Prevalence of pocket presence stratified by age within each KL grade

Pocket prevalence was further examined by age decade within each KL grade. No consistent pattern across age groups was observed across KL grades 0–4. Age was not significantly associated with pocket presence within individual KL strata.

### Localization of the pocket in KL grade 0 knees

**Fig. 5:**
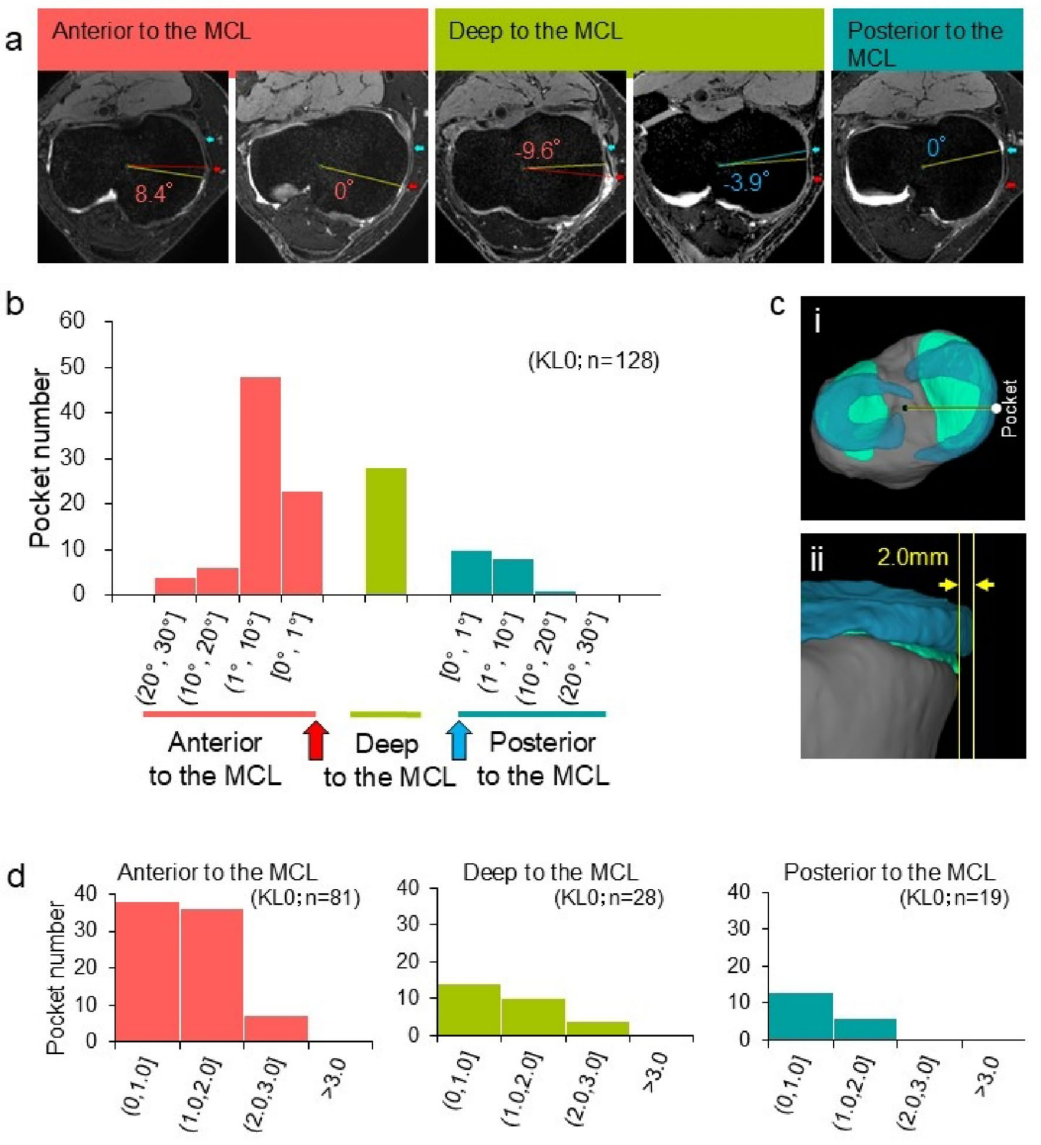
Localization of the pocket and its association with medial meniscal extrusion (MME) width in KL grade 0 knees. (**a**) Method for localizing the pocket and representative examples. We define the center of the tibia on axial MRI images. Based on the positional relationship between the pocket and the medial collateral ligament (MCL), we classify the pocket location into one of three groups: Anterior to the MCL, Deep to the MCL, or Posterior to the MCL. (The anterior edge of the MCL is indicated by a red arrow, and the posterior edge is indicated by a blue arrow.) In the Anterior to the MCL group, we express the localization as the angle between the posterior edge of the pocket and the anterior edge of the MCL. In the Deep to the MCL group, we use the same method, but the angle is represented as a negative value. In the Posterior to the MCL group, we measure the angle between the anterior edge of the pocket and the posterior edge of the MCL. (**b**) Distribution of pocket locations in KL grade 0 knees. The interval (0 °,10 °] represents values > 0 ° and ≤ 10 °. Among the KL grade 0 knees with a pocket sign (*n* = 120), 8 knees had two pockets (total pockets = 128). (**c**) Measurement of MME width at the pocket site. (i) For the 3D reconstruction of the tibial plateau, the model is rotated around the central axis so that the pocket center aligns with the lateral-most position, which standardizes the orientation for comparisons. (ii) A frontal 3D view is used to automatically quantify the MME width, defined as the horizontal distance from the medial edge of the tibial plateau to the outer margin of the medial meniscus at the pocket site. In the example shown here, the width is 2.0 mm. (**d**) Distribution of the MME width at the pocket site in KL grade 0 knees. The interval (0, 1.0) mm represents values > 0 and ≤ 1.0 mm.

Based on the positional relationship between the pocket and the medial collateral ligament (MCL), the pocket locations were classified into the following three groups: Anterior to the MCL, Deep to the MCL, and Posterior to the MCL (Fig. 5A). Among the KL grade 0 knees with a pocket sign (*n* = 120), 8 knees had two pockets, yielding a total of 128 pockets. No knee had three or more pockets. All 8 knees with two pockets exhibited the pattern of having one pocket Anterior to the MCL and one Posterior to the MCL. Among these 128 pockets, the significantly more pockets were noted in the Anterior to the MCL group (81/128, 63%) than in the Deep to the MCL group (28/128, 22%) or the Posterior to the MCL group (19/128, 15%) (*p* < 0.01) (Fig. 5B).

In the Anterior to the MCL group, the most common pockets (48 pockets) were those in which the posterior edge of the pocket was located 1° and ≤ 10° from the anterior edge of the MCL, without being in contact with it. The second most common pockets (28 pockets) were those in which the pocket was in the Deep to the MCL. In the Posterior to the MCL group, 10 pockets had the anterior edge of the pocket in contact with the posterior edge of the MCL [0°,1°).

### Pocket location and MME width in KL grade 0 knees

Quantification of the width of the medial meniscal extrusion (MME) at the pocket site (Fig. 5C-i, ii) yielded values ranging from > 0 mm to ≤ 3 mm across all three groups (Fig. 5D). In the Anterior to the MCL group, the majority of cases had MME width between > 0 mm and ≤ 2.0 mm.

### Intraoperative observation of the pocket sign

**Fig. 6:**
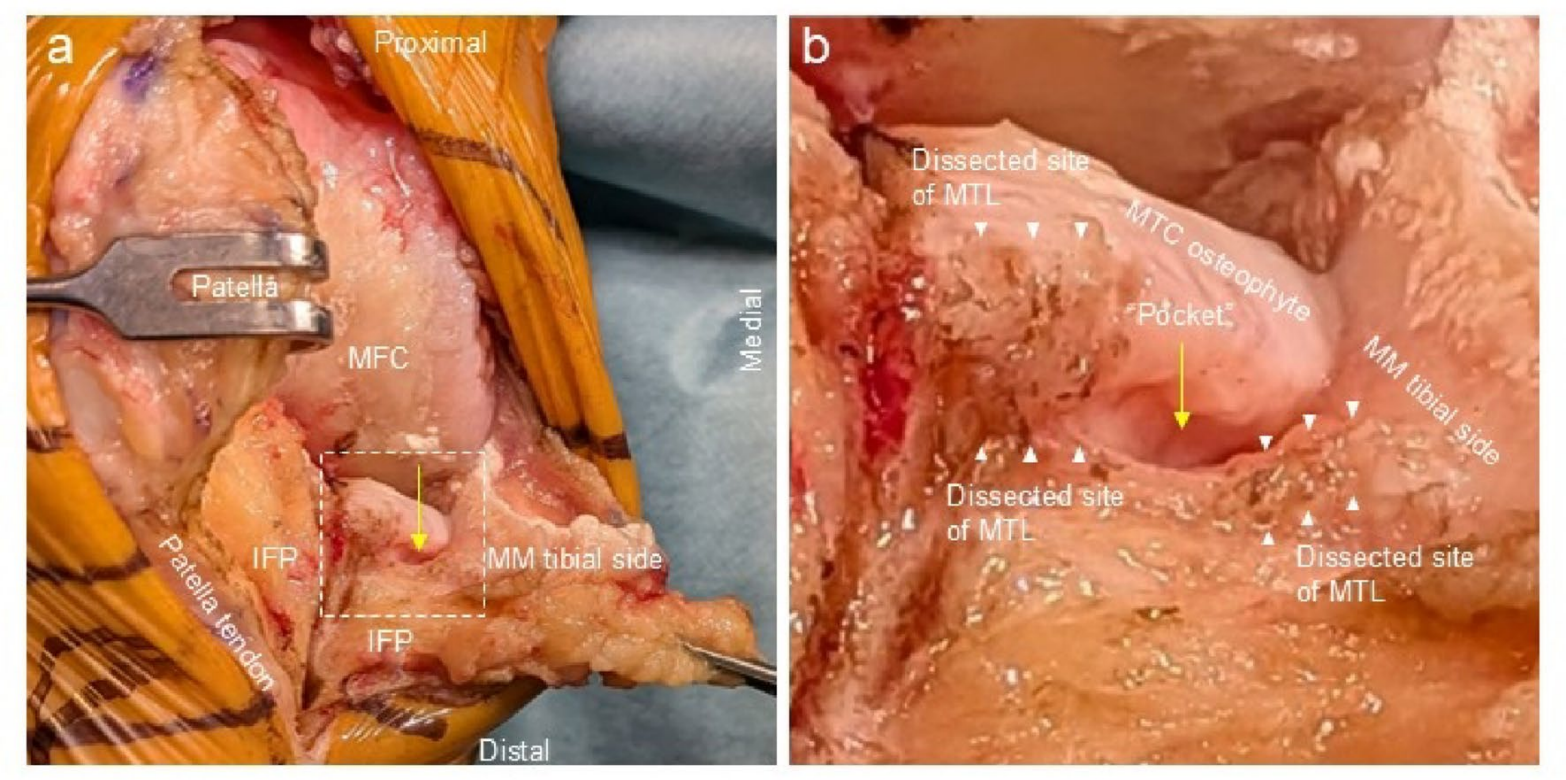
Intraoperative findings related to the pocket sign. (**a**) Intraoperative view during total knee arthroplasty in a patient with KL grade 3 medial knee OA of the right knee. A longitudinal medial incision was made along the patella and patellar tendon. Application of gentle distal–medial traction to the joint capsule and careful dissection using an electrosurgical device from the anterior aspect of the meniscotibial ligament revealed lack of apposition at the ligament–tibial interface, corresponding to the pocket sign. (**b**) Enlarged view of the white dotted square. The dissected site of the meniscotibial ligament is indicated by white arrowheads and shows signs of electrocautery charring. The pocket sign is indicated by yellow arrows. MFC, medial femoral condyle; IFP,  infrapatellar fat pad; MTC, medial tibial condyle; MM, medial meniscus.

During total knee arthroplasty , we attempted to identify findings at the meniscotibial ligament insertion corresponding to the pocket sign on MRI. Figure 6 shows a representative case. After making a longitudinal medial incision along the patella and the patellar tendon, we applied gentle distal–medial traction to the joint capsule and gently retracted the anterior horn of the medial meniscus distally and posteriorly. We then carefully dissected the meniscotibial ligament using an electrosurgical device from the anterior aspect. We observed a finding corresponding to the pocket sign posterior to the artificially dissected area, which was compatible with possible pre-existing detachment (Fig. 6A, B).

## Discussion

This study demonstrates that a separation-like appearance between the ligament attachment and the tibial cortex, visualized as the pocket sign on MRI, is associated with the radiographic severity of medial knee OA. The prevalence of the pocket sign was substantial even in knees without radiographic OA (KL grade 0) and increased progressively with higher KL grades. These findings indicate a strong cross-sectional association between pocket presence and structural disease severity.

The imaging protocol used in this study was designed to provide a systematic and reproducible method for identifying the pocket sign. By combining axial and coronal multiplanar reconstructions and by requiring visualization across consecutive sections, we aimed to minimize misclassification due to partial volume effects or joint effusion. For reliability assessment, Cohen’s κ coefficients were interpreted according to the criteria proposed by Landis and Koch, in which values of 0.61–0.80 indicate substantial agreement and values greater than 0.80 indicate almost perfect agreement^12^. The observed κ values for both intra- and inter-observer reliability indicated substantial agreement, supporting the reproducibility of this imaging definition. This standardized approach may facilitate future investigations of meniscotibial ligament morphology in large imaging cohorts.

The presence of both pocket-positive and pocket-negative knees across KL grades highlights the heterogeneity in meniscotibial ligament status. Importantly, the pocket sign was not observed in all knees with structural abnormalities, indicating that this MRI finding may reflect only one aspect of the structural heterogeneity of knee OA. The coexistence of structurally intact ligaments in some knees with radiographic changes suggests that meniscotibial ligament failure may develop in parallel with—rather than as an inevitable consequence of—other joint structural alterations. These observations are consistent with an emerging viewpoint that knee OA comprises diverse structural phenotypes rather than embodying a single uniform degenerative process, with meniscotibial ligament dysfunction potentially representing one such structural subtype^13^.

The observed increase in pocket prevalence across KL grades indicates a strong association between the pocket sign and radiographic severity. In KL grade 0 knees, pocket signs were identified in approximately 40% (120/308 knees) of the cases, suggesting this MRI finding can be detected in the absence of radiographic OA. However, because this was a cross-sectional study, no inference can be made regarding the temporal sequence between pocket formation and radiographic changes. Clarification of whether pocket formation precedes, accompanies, or follows other structural alterations will require further longitudinal studies.

The anterior predominance of pocket location relative to MCL indicates a nonrandom anatomical distribution. Most anterior pockets were located within 0–10° of the anterior MCL edge, whereas posterior pockets consistently contacted the posterior MCL edge. This distribution pattern may reflect regional differences in local anatomy or mechanical loading; however, the present data do not allow any mechanistic conclusions. Rather, the findings provide an anatomical context for future biomechanical or longitudinal investigations.

The anatomical findings of Yoshida et al.^14^ are consistent with our observation of anterior predominance. Their study demonstrated that the medial meniscotibial ligament attachment is deepest at the 2 o’clock position, corresponding to our anterior group. This anatomical configuration provides structural context for the observed distribution pattern. While cadaveric studies measure fixed anatomical distances, MRI detects fluid signals at the ligament–bone interface in living tissue. These methodological differences underscore the importance of using systematic imaging criteria to distinguish normal anatomical recesses from findings corresponding to the pocket sign.

One possible biomechanical interpretation of the anterior predominance observed in the present study relates to stress distribution during knee flexion^15^. As the medial meniscus translates posteriorly, regional differences in loading patterns across the meniscotibial ligament may occur. Experimental studies have suggested that repetitive tensile strain can influence ligament structure^16^. However, as the present cross-sectional data do not allow confirmation of any specific mechanical mechanism, these considerations should be regarded as hypotheses for future biomechanical investigation rather than causal explanations.

The association between pocket presence and MME in KL grade 0 knees further supports a structural relationship between ligament attachment status and meniscal position. Measurable MME was observed at pocket sites even in radiographically normal knees. Nevertheless, because MME was quantified only in pocket-positive KL grade 0 knees, direct comparison with pocket-negative knees was not possible using this protocol. Accordingly, the strength and directionality of the relationship between detachment-related MRI findings and meniscal displacement require further evaluation.

Intraoperative visualization during total knee arthroplasty provided limited supportive correlation with MRI findings, as lack of apposition between the meniscotibial ligament and the tibial cortex was observed in cases with pocket signs. However, the limited number of intraoperative cases precludes definitive validation, and these observations should be interpreted cautiously.

**Fig. 7:**
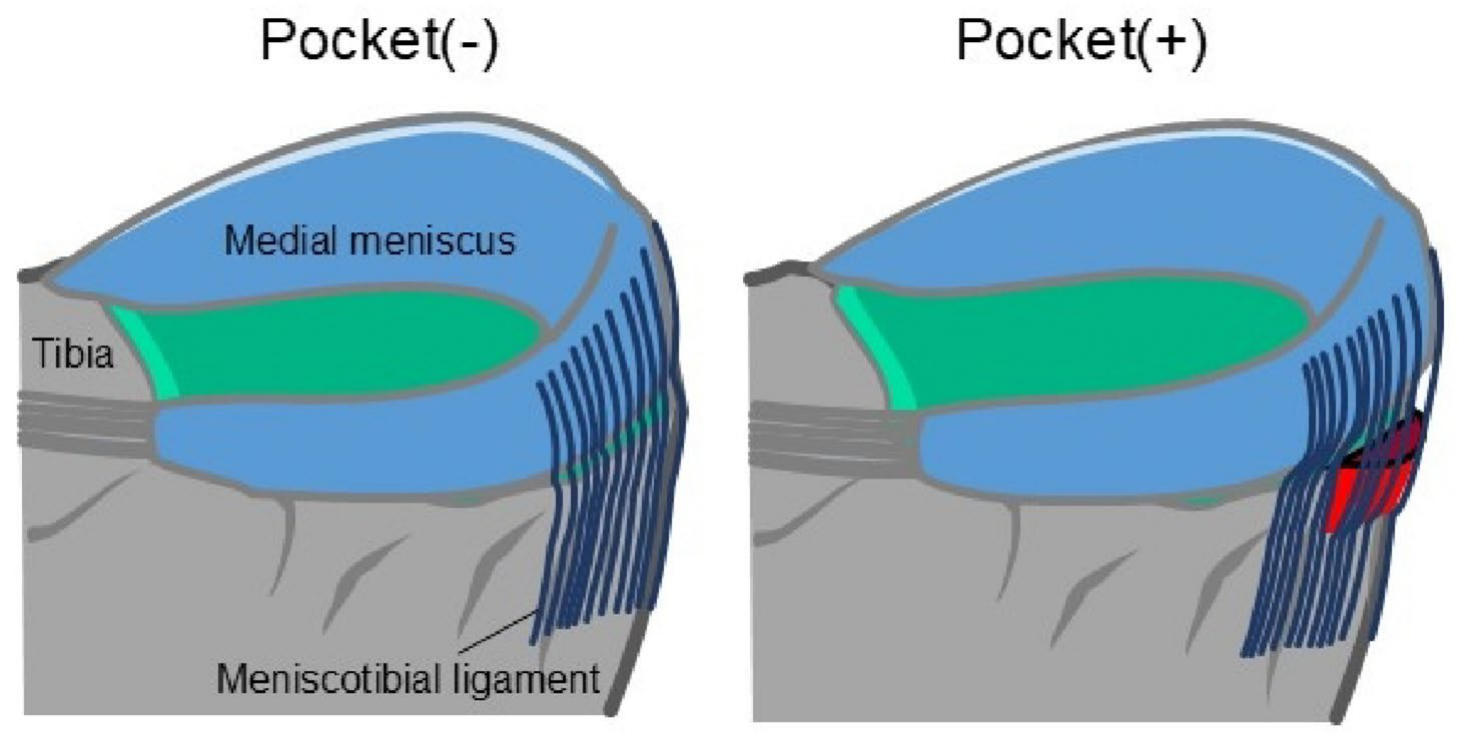
Schematic illustration of meniscotibial ligament attachment status with and without the pocket sign. Left (Pocket −): The meniscotibial ligament is normally attached to the medial tibial cortex, maintaining stable anchorage of the medial meniscus to the tibia. Right (Pocket +): Lack of apposition between the deep meniscotibial ligament and the tibial cortex creates a potential space between them (red area). Joint fluid may accumulate within this space, appearing as a “pocket sign” on MRI.

We provide a schematic illustration (Fig. 7) depicting the attachment status of the meniscotibial ligament in pocket-negative and pocket-positive knees. In pocket-negative knees, the ligament is shown as firmly attached to the medial tibial cortex, where it maintains stable anchorage of the medial meniscus. In contrast, in pocket-positive knees, the lack of apposition creates a potential space at the ligament–tibial interface. This potential space may allow the accumulation of joint fluid, which corresponds to the pocket sign observed on MRI.

 The pocket sign should be distinguished from fluid within the MCL bursa^17^. The MCL bursa lies between the superficial and deep layers of the MCL and may become visible on MRI when distended with fluid. In contrast, the pocket sign is characterized by a focal fluid signal in direct contact with the medial tibial cortex at the meniscotibial ligament–bone interface. On MRI, MCL bursal fluid typically appears as a smooth collection between ligament layers^18^, whereas pocket signals are localized at the ligament attachment site. These anatomical distinctions support the use of strict imaging criteria when identifying pocket signs^19^.

Several limitations of this study should be acknowledged. First, the cross-sectional design precludes conclusions regarding causality or temporal relationships between pocket formation and radiographic severity. Second, because lower limb alignment was not evaluated, it was not included in the multivariable model, and this may have influenced the observed associations. Third, because the MME width was quantified only in pocket-positive KL grade 0 knees, this precluded any direct comparison with pocket-negative knees and limited interpretation of the association between the presence of the pocket sign and meniscal position. Prospective longitudinal studies incorporating alignment assessment, biomechanical evaluation, and standardized intraoperative validation are warranted to provide further clarification of the clinical relevance of the pocket sign.

In conclusion, the pocket sign on MRI, reflecting changes at the ligament insertion, including possible detachment, is strongly associated with radiographic severity of medial knee OA. The finding is prevalent even in KL grade 0 knees and demonstrates a characteristic anterior anatomical distribution. These results establish the pocket sign as a reproducible MRI feature associated with structural joint changes and provide a foundation for future longitudinal investigation.

## Data Availability

The imaging datasets contain protected health information and are not publicly available due to ethical and legal restrictions. Qualified investigators at not-for-profit institutions may request access to de-identified, limited datasets for pre-specified, IRB-approved purposes under a Data Use Agreement. Contact the corresponding author.
